# Prevalence and correlates of body dysmorphic disorder and its association with social media use among high school students in Abha city, Aseer region, Saudi Arabia

**DOI:** 10.1097/MD.0000000000047815

**Published:** 2026-02-28

**Authors:** Abdulaziz Muflih Abudasser, Abdulaziz Mohammad Al-Garni, H.S. Alamri, Waddah Alalmaei Asiri, Abdulmajeed A. Zarbah, Amal Saad Alshahrani, Fatima Ahmed Badawi, Ghaidaa Abdulrahman Alqahtani, Osama Ayed Saleh Asiri, Hanan Delem Almoghamer, Abdulelah Nasser Shaya Alghaeb, Bandar Musharraf A. Alamri, Khaled Mohammed Hasan Asiri

**Affiliations:** a Department of Medicine, College of Medicine, King Khalid University, Abha, Kingdom of Saudi Arabia; b Department of Psychiatry, College of Medicine, Imam Abdulrahman Bin Faisal University, Dammam, Saudi Arabia; c College of Medicine, King Khalid University, Abha, Kingdom of Saudi Arabia.

**Keywords:** adolescents, BDD, mental health, Saudi Arabia, social media

## Abstract

This study aimed to assess the prevalence and correlates of body dysmorphic disorder (BDD) and to explore its association with social media usage among high school students in Abha city, Aseer region, Saudi Arabia. A cross-sectional study was conducted in the selected public high schools in Aseer region, Kingdom of Saudi Arabia, from March 12, 2023 to March 17, 2023. A total of 346 (36%) students were worried about how their look. The most areas of concern included their nose shape and size (26.7%), having acne (19.1%), abdomen shape (18.6%), and their weight (18.2%). Female students showed 3.5 times more likelihood for perceived BDD than males (OR = 3.52; 95% CI: 2.13–5.83), while non-Saudi showed 74% more likelihood for perceived BDD than Saudi students (OR = 1.74; 95% CI: 1.02–2.99). BDD is common in Saudi Arabia. Certain demographics were associated with BDD in our study such as female gender and non-Saudi Arabian nationality. The results emphasize the need for safe social networking, particularly among teenagers, and for public education about the dangers of borderline personality disorder and its effects.

## 1. Introduction

Body dysmorphic disorder (BDD) is a psychological condition characterized by an obsessive preoccupation with 1 appearance, coupled with an overwhelming fear of perceived flaws or imperfections that may go unnoticed by others. It is vital to emphasize that BDD should not be diagnosed in the presence of objectively observable physical defects. Those afflicted by BDD endure persistent distress, anxiety, and melancholy due to their appearance, leading to substantial impairments in their daily functioning.^[1]^ Their unshakable belief in the existence of a severe aesthetic flaw renders them fearful of being considered ugly or disfigured, causing them considerable emotional distress.^[2–4]^

Exploring the existing body of literature, the precise risk factors associated with BDD remain somewhat elusive. Nevertheless, several factors are postulated to contribute to the development of BDD. These include genetic predisposition, early-life adversity, such as experiences of bullying or teasing, and the absence of familial support.^[2–4]^ Additionally, instances of sexual abuse might serve as nonspecific factors in this complex equation, as may an increased sensitivity to aesthetic aspects beyond the average.^[2]^ It is essential to recognize that these potential risk factors, when intertwined with a person susceptibility to BDD, can create a challenging landscape for understanding and addressing this condition.

In 2016, a comprehensive review was conducted to investigate the prevalence of BDD within the general population. This data unveiled a compelling insight into the extent of BDD presence among people who do not necessarily seek clinical or psychiatric help for their condition. The results of this analysis indicated that, on average, approximately 1.9% to 2.2% of individuals in the general population are affected by BDD. To put this into perspective, BDD is a psychological condition characterized by an obsessive focus on perceived flaws or defects in 1 appearance, which often leads to significant distress and impairment in daily functioning.^[5]^

In Saudi Arabia, the reported incidence of BDD among the general population stands higher, ranging from 4.2% to 8.8%, and among female students, it escalates to 4.4% to 12.3%.^[6–9]^ Conversely, when focusing on nonclinical settings, such as college campuses, the prevalence of BDD exhibits variability, spanning from 1.3% to 5.8%. Among college students, the lower end of this spectrum is seen in Chinese students (1.3%) and American students (4%). In contrast, a more elevated prevalence of BDD is observed in Pakistani, Turkish, and German college students, where the rates reach 5.8%, 4.8%, and 5.3%, respectively.^[10–14]^

Over the past decade, social media has exhibited an exponential penetration into the daily lives of individuals. Arab youths were among the most active communities on social media usage. Although youth believe social media injects so much happiness and excitement into their lives.

To date, few studies in Saudi Arabia have explored the prevalence of BDD among adolescents and its relation to social media exposure.^[7,15]^ This study is among the 1st to investigate this association specifically among high school students in the Aseer region, a population that has not been previously studied in this context. The objective of the present study was to examine the prevalence, factors, and impacts of BDD among school students in Saudi Arabia and its relation to social media usage.

## 2. Methods

### 2.1. Study design and setting

A cross-sectional analytical study was conducted in public high schools in Abha city, located in the Aseer region of the Kingdom of Saudi Arabia, from March 12, 2023 to March 17, 2023. The study was conducted in accordance with the Declaration of Helsinki, STROBE guideline and approved by the Institutional Review Board of King Khalid University (approval number ECM#2022-1901).

### 2.2. Participants, sample size, and sampling techniques

The minimum required sample size was calculated to be 750 students, based on an estimated BDD prevalence of 5%, a margin of error of 2%, a confidence level of 95%, and a design effect of 2, assuming a total accessible population of 2000 students. A multistage cluster sampling technique was employed. Initially, a list of public high schools in Abha city was obtained from the Ministry of Education. The city was divided into 5 administrative zones: central, east, west, north, and south. From each zone, 2 schools were selected using a simple random sampling method. Within each selected school, students were chosen randomly with the help of a student guide or class roster to ensure equal representation across grades and sexes.

Inclusion criteria: age ≥ 15 years old, both sexes and currently enrolled in secondary school in Abha in Saudi Arabia.

Exclusion criteria included: diagnosis of any known psychiatric disorder under treatment and incomplete or missing responses on the key study questionnaires.

### 2.3. Tools for data collection

Data were collected using the Body Dysmorphic Disorder Questionnaire (BDDQ), a self-rated screening tool that has demonstrated high diagnostic accuracy, with a sensitivity of 94%, specificity of 90%, and a likelihood ratio of 9.4 when compared with the Structured Clinical Interview for DSM-IV. BDDQ^[16]^ was used to assess BDD symptomatology among participants and used in previous studies.^[17,18]^ To ensure cultural and linguistic relevance, the original English version of the BDDQ was translated into Arabic following a standard forward–backward translation protocol.^[6]^ First, 2 bilingual experts independently translated the instrument from English to Arabic. Their translations were then combined into a single version through discussion. This version was back-translated into English by 2 other independent bilingual professionals who had no prior exposure to the original version. Any discrepancies were reviewed and resolved through consensus by a panel of experts, including psychologists and Arabic language specialists.

The translated questionnaire underwent pilot testing with 50 high school students (excluded from the main study) to assess clarity, comprehension, and internal consistency. Content validity was evaluated by a panel of 3 clinical psychologists familiar with adolescent mental health and BDD, who confirmed that the items were relevant and appropriate for the target population in Arabic language. Cronbach alpha for internal consistency was 0.82, indicating good reliability.

### 2.4. Data collection technique

After obtaining Institutional Review Board approval, permission was sought and obtained from the Ministry of Education in Aseer region to access public high schools in Abha city. Researchers personally visited the selected schools and obtained verbal consent from school administrators and class teachers to approach students. Similarly, verbal permission was obtained from all students. Moreover, verbal consent was also obtained from parents if the participating student age was <16 years old before the interview after explaining the study purpose and confidentiality measures. Data collection was carried out during regular school hours in classrooms, and students completed the BDDQ under supervision. No identifying information was collected, and students were allowed to skip questions they were uncomfortable answering.

### 2.5. Data analysis

Data were analyzed using SPSS version 16.0 (SPSS Inc., Chicago). Descriptive statistics, including frequencies and percentages, were used to summarize demographic and behavioral characteristics. Chi-square tests were applied to examine associations between categorical variables, including the relationship between BDD and gender, nationality, academic year, and social media usage.

The duration of social media use was categorized (e.g., <1, 1–3, >6 hours daily), and its association with perceived BDD was assessed. A significance level of 0.05 was used for all inferential statistics. Multivariable odds ratios and 95% confidence intervals were also calculated to assess the strength of associations.

## 3. Results

A total of 961 high school students (mean age 16.7 ± 2.1 years, range 15–20) participated. Most were female (59.3%) and Saudi nationals (88.8%). Regarding grade level, 32.2% were in the 1st year, 36% in the 2nd year, and 31.8% in the 3rd year. Academic performance was reported as excellent by 85.4%, very good by 12.7%, and good by 1.9%. About 70.8% engaged in sports, with 23.2% doing so once, 14.3% twice, and 14.8% 5 or more times weekly.

Appearance concerns were reported by 36%, and among 267 students preoccupied with their looks, 66.3% feared not being thin enough (Table 1). Top concerns included the nose (26.7%), acne (19.1%), abdomen (18.6%), weight (18.2%), breast size (17.4%), and hair (13.6%).

**Table 1:** Body dysmorphic disorder (BDD) criteria among study high school students, Aseer region, Saudi Arabia.

BDD criteria	No.	%
Are you worried about how you look?		
* *No	615	64.0
* *Yes	346	36.0
If yes: Do you think about your appearance problems a lot and wish you could think about them less? (n = 346)		
No	79	22.8
Yes	267	77.2
Is your main concern with how you look that you aren’t thin enough or that you might get too fat? (n = 267)		
No	90	33.7
Yes	177	66.3
What are these concerns? (n = 267)		
Nose	63	26.7
Acne	45	19.1
Abdomen	44	18.6
Weight	43	18.2
Breast	41	17.4
Hair	32	13.6
My skin	27	11.4
Height	21	8.9
Buttocks	21	8.9
Face	11	4.7
Teeth	9	3.8
All my body	2	0.8
Has this problem often upset you a lot? (n = 267)		
No	59	22.1
Yes	208	77.9
Has it often gotten in the way of doing things with friends, dating, your relationships with people, or your social activities? (n = 267)		
No	138	51.7
Yes	129	48.3
Has it caused you any problems with school, work, or other activities? (n = 267)		
No	173	64.8
Yes	94	35.2
Are there things you avoid because of how you look? (n = 267)		
No	171	64.0
Yes	96	36.0
On an average day, how much time do you usually spend thinking about how you look? (n = 267)		
<1 h a day	149	55.8
1–3 h a day	78	29.2
>3 h a day	40	15.0

BDD = body dysmorphic disorder.

Of those concerned, 77.9% reported emotional distress, 48.3% social interference, 35.2% academic/work interference, and 36% activity avoidance. Time spent thinking about appearance was under 1 hour in 55.8%, 1 to 3 hours in 29.2%, and over 3 hours in 15%.

Based on the BDDQ, 11.6% met criteria for BDD. BDD was more common among females (16% vs 5.1%, *P* = .001), non-Saudis (16.7% vs 10.9%, *P* = .049), and 3rd-year students (15% vs 12% in the 1st year and 8.1% in 2nd-year, *P* = .021). No other demographic or behavioral variables were significant (Table 2).

**Table 2:** Factors associated with students perceived body dysmorphic disorder (BDD).

Factors	Perceived BDD				*P* value
	No perceived BDD		Perceived BDD		
	No.	%	No.	%	
Age (yr)					.143
15–	130	89.0	16	11.0	
16–	283	89.3	34	10.7	
17–	299	85.7	50	14.3	
18+	138	92.6	11	7.4	
Gender					.001*
Male	371	94.9	20	5.1	
Female	479	84.0	91	16.0	
Nationality					.049*
Saudi	760	89.1	93	10.9	
Non-Saudi	90	83.3	18	16.7	
Study year					.021*
1st secondary year	272	88.0	37	12.0	
2nd secondary year	318	91.9	28	8.1	
3rd secondary year	260	85.0	46	15.0	
Academic performance					.944
Good	16	88.9	2	11.1	
Very good	109	89.3	13	10.7	
Excellent	725	88.3	96	11.7	
Do you practice sports?					.311†
Never practiced sports	245	87.2	36	12.8	
Once per week	194	87.0	29	13.0	
Twice per week	121	88.3	16	11.7	
3 times/wk	102	87.2	15	12.8	
4 times/wk	54	88.5	7	11.5	
5 times/more/wk	134	94.4	8	5.6	
Frequency of practicing sports					.327
Never	245	87.2	36	12.8	
1–2 times/wk	315	87.5	45	12.5	
3 times or more/wk	290	90.6	30	9.4	

*P*: Pearson X^2^ test.

BDD = body dysmorphic disorders.

† Exact probability test.

* *P* < .05 (significant).

**Figure 1.**
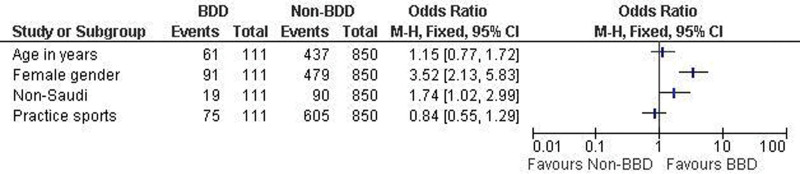
Regression analysis to identify factors associated with BDD among study students. BDD = body dysmorphic disorder.

Females had higher odds of BDD (OR = 3.52; 95% CI: 2.13–5.83), as did non-Saudis (OR = 1.74; 95% CI: 1.02–2.99) (Fig. 1).

While platform choice (TikTok, Snapchat) was not significantly linked to BDD, 33.3% of BDD students used social media ≥6 hours/day versus 25.2% without BDD; 20.7% used it 5 to 6 hours versus 13.5% without BDD (*P* = .027) (Table 3).

**Table 3:** Relation between perceived body dysmorphic disorders (BDD) and students use of social media.

Social media	Perceived BDD				*P* value
	No perceived BDD		Perceived BDD		
	No.	%	No.	%	
Frequently used social media platforms					.397
Tiktok	676	79.5	93	83.8	
Snapchat	665	78.2	84	75.7	
Instagram	533	62.7	80	72.1	
WhatsApp	435	51.2	53	47.7	
Telegram	298	35.1	41	36.9	
Twitter	273	32.1	44	39.6	
YouTube	499	58.7	66	59.5	
Facebook	21	2.5	1	.9	
Other	63	7.4	8	7.2	
How many hours do you spend per day on social media?					.027*
<1 h	26	3.1	2	1.8	
1–<2 h	79	9.3	4	3.6	
2–<3 h	124	14.6	8	7.2	
3–<4 h	139	16.4	17	15.3	
4–<5 h	153	18.0	20	18.0	
5–<6 h	115	13.5	23	20.7	
6 h/more	214	25.2	37	33.3	

*P*: Exact probability test.

BDD = body dysmorphic disorders.

* *P* < .05 (significant).

## 4. Discussion

In the present study, 961 high school students were included to observe the prevalence, related issues, and impacts of BDD-related symptomatology among school students in Saudi Arabia and its relation to social media usage. The present study shows that 11.6% of the study students fulfilled BDD-related symptomatology criteria among high school students in the Aseer region, Saudi Arabia.

While BDD is frequently encountered in clinical settings, numerous studies have revealed a notable prevalence of BDD among adult students in nonclinical environments.^[19,20]^ Several studies have shown that BDD is more prevalent among students, and varies widely by country, for example, 4.8% of Turkish college students,^[14]^ 5.3% of German college students,^[12]^ 5.8% of medical students in Pakistan,^[11]^ 13.9% of university students in Saudi Arabia,^[20]^ and 4.9% of university students in the USA.^[21]^

The student population spanned ages from 15 to 20 years, with 59.3% of them being female. Among these, 32.2% were in their 1st year, 36% were in their 2nd year, and 31.8% were in their 3rd year in the present study. A similar study featured an equivalent male-to-female student ratio and age range, as mentioned in this study.^[15]^ In contrast, another study exclusively encompassed female medical students from the 1st to the 5th year.^[8]^

The data shows that perceived BDD-related symptomatology is fairly evenly distributed among different age groups. Result that aligns with supporting findings from previous studies examined in a systematic review, reported a higher proportion of females was observed among adults, with a ratio of 1.27. Among students, there was an even stronger female predominance, with a ratio of 1.64.^[5]^ Due to the central role of beauty in the stereotypical perception of the female gender, women bodies are frequently subjected to evaluative scrutiny.^[22]^

The higher rate of BDD-related symptomatology among females compared to males can be linked to society beauty standards, social media exposure, and cultural influences. Women often face greater pressure to meet beauty ideals, leading to more self-criticism and body dissatisfaction.^[23]^ Social media plays a big role, as females are more likely to see edited images, beauty filters, and idealized body types, which can increase unrealistic expectations and insecurities.^[24]^ In Saudi Arabia, cultural factors may add to this issue, as physical appearance is often emphasized in social settings, making some women more conscious about their looks.^[6]^ Hormonal and emotional differences also play a part, as women are more likely to experience anxiety and stress related to body image, increasing their risk of BDD and other mental health issues.^[5]^

A similar study by Sindi AS, reported that the BDD population had an equal gender ratio and most included respondents between 18 and 27 years old (78.6%), and college students (60.7%) with no gender predominance,^[15]^ which is consistent with what was published in 2020 by The General Authority of Statistics in Saudi Arabia, in which the majority of the Saudi society is between 15 and 34 years old.^[25]^

The findings from our research align with those of previous studies, and this similarity may be attributed to the closely matched average ages in our study group. It is worth noting that younger individuals often exhibit a heightened preoccupation with their appearance compared to older generations, likely stemming from the physical and psychological transformations they undergo during this stage of life.

Preoccupying thoughts about 1 appearance can severely hinder concentration on school tasks, resulting in test failures. However, there is recorded evidence linking BDD to functional impairments, such as a decline in academic achievements, school disengagement, and eventual dropout.^[26,27]^ Moreover, BDD can disrupt an individual capacity to engage with peers and educators and, in some cases, can discourage students from attending school altogether. A study revealed that a staggering 18% of students suffering from BDD withdrew from school due to the severity of their symptoms.^[26]^

Supporting the present result, a study has shown that BDD was more prevalent among individuals who had pursued aesthetic treatments and those who had a sedentary lifestyle.^[28]^ The competitive and highly critical nature of sports training and competition often leads to a negative self-image, and making changes to 1 body image is seen as a way to enhance self-esteem and self-evaluation.^[29]^

Present study also highlighted that among those with appearance-related concerns, 66.3% were primarily worried about being thin enough or the fear of getting too fat. This finding lines up with body image and weight-related issues that are commonly associated with BDD. Besides, specific concerns related to the appearance, such as the nose, acne, abdomen, and weight, were identified among this group. These concerns often lead to significant distress.

Furthermore, individuals experiencing BDD may exhibit anxieties regarding various aspects of their appearance, with particular emphasis on features like the nose, eyes, skin, muscles, and hair,^[30]^ although it is not uncommon for people to have some degree of concern about their physical appearance. Nonetheless, when these concerns lead to personal distress and hinder an individual ability to engage effectively in social and occupational contexts, regardless of whether the perceived flaws are real or imagined, it is indicative of a condition recognized as body dysmorphic disorder.^[31]^ The most reported areas of concern among students with BDD-related symptomatology were nose shape and size followed by breast shape in the present study. In contrast, another study reported that those with no BDD are more likely to be bothered by the shape or size of the belly than those with BDD.^[6]^ However, it is important to note that social media can exert a detrimental influence on individuals body image satisfaction, as indicated by several studies.^[32–34]^

By doing a comparative analysis of the participants with BDD-related symptomatology and concerning the duration of social media usage, it was observed that a significant majority of the participants (64.28%) allocated >4 hours a day to social media, while 30.95% spent between 2 and 4 hours daily on these platforms.^[35]^ In addition, TikTok, Snapchat, and Instagram were the most worryingly used social media applications in the present study, as revealed by the duration spent. Interestingly, BDD was significantly associated with a longer duration spent on applications. This could suggest a greater desire among individuals with BDD to share and keep tabs on their photos and videos. Snapchat, in particular, is known for its features that allow the editing and customization of personal photos and videos, which aligns with common behaviors observed in individuals with BDD.^[31]^ The study participants’ worrisome use of multiple social media apps may suggest a concern about missing out on possibly fulfilling experiences that others are enjoying.^[36]^ The heavy use of social media, together with its negative impact on body image, may explain the higher rates of BDD in the present study.

There is a suggestion from the previous studies, that social media may exacerbate negative body image beliefs by promoting universally accepted beauty standards.^[32–34]^ Disseminating images, clips, and remarks concerning the ideal facial features, nasal structure, hair qualities, and skin tone can readily incite the notion that an individual bodily attributes diverge from the norm. In alignment with this suggested connection, individuals with BDD in the present research exhibited a higher tendency to measure their looks against prominent figures in the realm of social media, that why they give a lot of importance to how they look and focus on what they think is different about their body.^[6]^

### 4.1. Limitations

This study has several limitations that should be considered when interpreting the findings. First, the study was conducted exclusively among high school students in the Aseer region of Saudi Arabia, which may limit the generalizability of the results to the broader Saudi population. Differences in urban versus rural environments, socioeconomic status, and access to and patterns of social media usage across different regions of the Kingdom may influence the prevalence and factors associated with BDD. Therefore, caution should be exercised when applying these results to all Saudi youth or the general public. Second, although a validated screening tool (BDDQ) was used, the diagnosis of BDD was based on self-reported responses, not confirmed clinical interviews. This may introduce response bias or over- or underestimation of BDD prevalence. Third, the cross-sectional design of the study prevents establishing causality between social media usage and the development of BDD symptoms. Longitudinal studies would be better suited to assess temporal relationships. Fourth, while this study categorized social media use based on daily hours spent on platforms, it did not assess the nature of engagement, such as passive scrolling, active posting, or exposure to beauty-related content. Fifth, while the survey included general indicators of psychological health (such as stress, anxiety, and depression), it did not use psychometric tools specifically designed to measure the correlation between these factors and BDD symptoms. Therefore, we could not assess comorbidity patterns or psychological predictors of BDD. Finally, although we assessed the duration of social media use, we did not explore the qualitative nature of social media engagement, such as the type of content consumed (e.g., beauty influencers, filtered images) or the emotional impact of interactions online. These qualitative dimensions may have provided deeper insights into the link between social media exposure and body dissatisfaction. Despite these limitations, the study provides important preliminary data on BDD among high school students in Saudi Arabia and highlights areas for further research and preventive strategies.

### 4.2. Practical implication

The findings of this study have important practical implications. The significant association between higher social media use and BDD symptoms highlights the need for school-based mental health awareness campaigns and early screening programs for adolescents. Educational initiatives targeting healthy social media behaviors, body positivity, and emotional resilience could help mitigate the risk of body image disturbances among youth. Additionally, the study supports the integration of digital literacy programs into school curricula to raise awareness about the unrealistic beauty standards often portrayed on social media platforms. These strategies can guide educators, healthcare professionals, and policymakers in developing culturally sensitive interventions aimed at promoting psychological well-being in Saudi adolescents.

## 5. Conclusion

BDD was found to be relatively common among high school students in Abha city, Aseer region, Saudi Arabia. Female gender, non-Saudi nationality, and extended social media use were significantly associated with higher BDD prevalence. These findings underscore the importance of promoting safe and mindful social media engagement among adolescents and the need for early educational interventions to raise awareness about the psychological effects of excessive exposure to appearance-focused online content.

## Author contributions

**Conceptualization:** Abdulaziz Muflih Abudasser, H.S. Alamri, Waddah Alalmaei Asiri, Abdulmajeed A. Zarbah.

**Data curation:** Abdulaziz Muflih Abudasser.

**Formal analysis:** Abdulaziz Muflih Abudasser.

**Investigation:** Abdulaziz Muflih Abudasser, Abdulaziz Mohammad Al-Garni.

**Methodology:** Abdulaziz Muflih Abudasser.

**Supervision:** Abdulaziz Muflih Abudasser, H.S. Alamri.

**Writing – original draft:** Abdulaziz Muflih Abudasser, Abdulaziz Mohammad Al-Garni, H.S. Alamri, Waddah Alalmaei Asiri, Abdulmajeed A. Zarbah, Amal Saad Alshahrani, Fatima Ahmed Badawi, Ghaidaa Abdulrahman Alqahtani, Osama Ayed Saleh Asiri, Hanan Delem Almoghamer, Abdulelah Nasser Shaya Alghaeb, Bandar Musharraf A Alamri, Khaled Mohammed Hasan Asiri.

**Writing – review & editing:** Abdulaziz Muflih Abudasser, Abdulaziz Mohammad Al-Garni, H.S. Alamri, Waddah Alalmaei Asiri, Abdulmajeed A. Zarbah, Amal Saad Alshahrani, Fatima Ahmed Badawi, Ghaidaa Abdulrahman Alqahtani, Osama Ayed Saleh Asiri, Hanan Delem Almoghamer, Abdulelah Nasser Shaya Alghaeb, Bandar Musharraf A Alamri, Khaled Mohammed Hasan Asiri.

**Resources:** Amal Saad Alshahrani, Fatima Ahmed Badawi, Ghaidaa Abdulrahman Alqahtani, Osama Ayed Saleh Asiri, Hanan Delem Almoghamer, Abdulelah Nasser Shaya Alghaeb, Bandar Musharraf A Alamri, Khaled Mohammed Hasan Asiri.
